# Application methods of tracers for N_2_O source determination lead to inhomogeneous distribution in field plots

**DOI:** 10.1002/ansa.202000100

**Published:** 2020-09-23

**Authors:** Jacqueline Berendt, Arne Tenspolde, David Rex, Tim J. Clough, Nicole Wrage‐Mönnig

**Affiliations:** ^1^ Grassland and Fodder Sciences Faculty of Agricultural and Environmental Sciences University of Rostock Rostock Germany; ^2^ Soil and Physical Sciences Department Faculty of Agriculture and Life Sciences Lincoln University Lincoln New Zealand

**Keywords:** ^15^N, bromide, nitrous oxide, spraying, stable isotope, syringe, watering can

## Abstract

Source determination of N_2_O has often been performed using stable isotope incubation experiments. In situ experiments with isotopic tracers are an important next step. However, the challenge is to distribute the tracers in the field as homogeneously as possible. To examine this, a bromide solution was applied as a stand‐in tracer using either a watering can, a sprayer, or syringes to a relatively dry (25% gravimetric moisture content) or wet (30%) silt loam. After 1 h, samples were taken from three soil depths (0‐10 cm), and analyzed for their water content and bromide concentration. The application with syringes was unsuccessful due to blocked cannulas. Therefore, further laboratory experiments were conducted with side‐port cannulas. Despite a larger calculated gravimetric soil moisture difference with watering can application, more Br^‐^ tracer was recovered in the sprayer treatment, probably due to faster transport of Br^‐^ through macropore flow in the wetter conditions caused by the watering can treatment. The losses of Br^‐^ (33% for the watering can, 28% for the sprayer treatment) are equivalent to potential losses of isotopic tracer solutions. For application of 60 at% ^15^NH_4_^+^, this resulted in theoretical enrichments of 44‐53 at% in the upper 2.5 cm and 7‐48 at% in 5‐10 cm. As there was hardly any NO_3_^‐^ in the soil, extrapolations for ^15^NO_3_^‐^ calculated enrichments were 57‐59 at% in the upper 2.5 cm and 26‐57 at% in 5‐10 cm. Overall, no method, including the side‐port cannulas, was able to achieve a homogeneous distribution of the tracer. Future search for optimal tracer application should therefore investigate methods that utilize capillary forces and avoid overhead pressure. We recommend working on rather dry soil when applying tracers, as tracer recovery was larger here. Furthermore, larger amounts of tracer lead to more uniform distributions. Further studies should also investigate the importance of plant surfaces.

## 1 INTRODUCTION

Nitrous oxide (N_2_O) is a long‐lived greenhouse gas with an average concentration of 324 ppb in the atmosphere.^1^ Since pre‐industrial times, this concentration has risen by about 19%.^2^ In the stratosphere, N_2_O is decomposed to active components involved in stratospheric ozone destruction.^3^, ^4^, ^5^ The main terrestrial sources of N_2_O production are natural soils and agricultural land.^6^ Nitrification and denitrification are the most important microbial pathways for the production of N_2_O in soil.^7^ However, there are many other microbial and chemical sources that are challenging to distinguish as they may take place simultaneously.^8^, ^9^, ^10^

To aid in the differentiation among these pathways, isotopic tracer methods have been developed.^8^ Commonly used isotopic tracer methods include the triple labeling approach^11^ and the dual‐isotope method.^12^ These methods are based on the addition of ^15^N (in form of NH_4_NO_3_ in the triple labeling) or of ^15^N and ^18^O tracers (in the dual isotope method). Both assume a homogeneous distribution of tracers in the soil. So far, these methods have usually been carried out in laboratory incubation experiments, where isotopic tracer solutions can be mixed into the soil. Future improvements in our understanding of N transformations in undisturbed soils requires that such experiments can also be performed in the field.

Previously, studies have applied isotopic tracers to intact soils using a variety of methods. Ideally, ^15^N tracer should be evenly distributed vertically and horizontally in the soil.^13^ To achieve this, Wang et al^14^ recommended a multi‐injector consisting of 10 syringes with side‐port cannulas, while Sgouridis et al^15^ injected ^15^N tracer with syringes fitted with normal cannulas (about 10 to 15 cm long). When labeling larger experimental plots in the field with ^15^N tracer, it is also important that the labeling can be performed rapidly. Accordingly, some studies have applied ^15^N tracer solutions using watering cans.^16^ However, it is not clear what depth of soil becomes labeled using such a technique, or how homogeneously the label is distributed.

Ionic charge on the ^15^N tracers may also cause bias in the ensuing distribution through the soil. For example, when using ammonium (NH_4_^+^) and nitrate (NO_3_^−^) as tracers, NH_4_^+^ may remain in the upper layers of the soil while NO_3_^−^ is displaced with water and possibly leached down the profile. Such a biased distribution of the NH_4_^+^ and NO_3_^−^ tracers could ultimately affect the calculation of N_2_O sources.

To date, the effects of different tracer application methods on the distribution of ^15^N tracers in field plots has not been studied. Therefore, we compared different methods for applying ^15^N tracers to soil using a bromide (Br^−^) solution as a stand‐in tracer and measuring gravimetric soil moisture content and soil Br^−^ concentrations as proxies for initial isotopic tracer distributions. The Br^−^ ion is considered a conservative tracer because it occurs at relatively small concentrations in natural soils and no chemical alterations take place when Br^−^ comes into contact with soil or water.^17^, ^18^ Due to the negatively charged Br^−^ ion, this experiment simulated more the distribution of NO_3_^−^. If a homogeneous distribution is not achieved for this, it will certainly not be achieved for NH_4_^+^.

We hypothesized that there would be differences among the application treatments using watering can, sprayer, or syringes concerning the time needed for application as well as the homogeneity of application. We hypothesized a trade‐off between time needed and homogeneity, with injection and watering can applications at the extremes. Furthermore, a higher run‐off is possible with the watering can, but we hypothesized that the solution might reach deeper soil layers more quickly than with the sprayer due to the larger hydraulic head. Although we expected larger run‐off, more dilution of the label and more macropore flow in wet than in dry soil, leading to less label recovery and more heterogeneity, we hypothesized that in general, the Br^−^ recovery would be linked to an increase in moisture content after application in both dry and wet soil. The observed Br^−^ tracer distribution was used to assess the theoretical distribution and enrichment of isotopic tracers.

## 2 MATERIALS AND METHODS

This experiment was performed at the experimental station of Lincoln University (43° 38′ 54.35″ S, 172 ° 28′ 06.01″ E), New Zealand, in November 2018, on a Wakanui silt loam (Mottled Immature PallicSoil [New Zealand classification^19^]; AericEpiaquept [USDA]). The grassland site, comprising of *Lolium perenne* and *Trifolium repens*, had not been grazed for 10 years, with a history of mowing over this time. The NH_4_^+^ content in the soil was 7.82 mg kg^−1^ whereas the NO_3_^−^ content was only 1.18 mg kg^−1^. The following treatments (n = 4) were compared: application of Br^−^ solution (2.5 L, equivalent to 10 mm of precipitation) by either (a) watering can, (b) hand‐held sprayer (hereafter called “sprayer”), or (c) injection with syringes. In addition, there were four controls without added tracer. The experimental design comprised a randomized complete block with 20 plots of 0.25 m² each (0.5 m × 0.5 m). Before applying the solution, the vegetation on all plots was cut to 10 cm by a lawn mower to minimize the interception of the solution by the plants.^16^ The experiment was performed on dry soil with an average gravimetric soil moisture content before application of 25.0% ± 1.4% (Experiment 1). In order to analyze the effect of the initial soil moisture on tracer homogeneity, the experiment was repeated with the watering can and control treatments after a rain event that delivered 65.6 mm precipitation over several days and resulted in the soil having an average gravimetric soil moisture content of 30.4% ± 3.4% (Experiment 2). In addition to that, a third experiment was also performed with a “brilliant blue” tracer dye applied by watering can on to the wet soil^20^ to visualize the penetration into the soil.

The application of Br^−^ solution (0.5 g Br^−^ L^−1^ as KBr) was calculated to achieve a soil Br^−^ concentration of ∼100 μg Br^−^ g^−1^ dry soil (assuming a uniform distribution to 10 cm depth) and thus ensure detectability. For Experiment 1 and 2, Br^−^ tracer solution was first applied as uniformly as possible to the whole plot using either the watering can or sprayer methods. Then the application of the Br^−^ tracer via syringe was performed in Experiment 1 in a linear pattern of seven rows with six injections per row, with the total solution volume for the plot being divided into 42 syringes of 60 mL each. The length of the cannula reached a depth of 3.8 cm and had an inner diameter of 0.8 mm (BD PrecisionGlide™ needle). This was shorter than used in a previous study:^15^ since we took samples 1 h after Br^−^ tracer application, we wanted to apply the Br^−^ tracer as homogeneously as possible to the upper soil layer to reach comparability with the other application methods.

One hour after Br^−^ tracer application, 10 soil samples were randomly taken from Experiment 1 and 2 with a soil corer (inner diameter 2.5 cm by 10.0 cm deep) from the inner part of the plot. These soil cores were divided into three depths: 0–2.5 cm, 2.5–5.0 cm, and 5.0–10.0 cm, hereafter referred to as depths 1, 2, and 3, respectively.

For Experiment 3, “brilliant blue” tracer dye was mixed with water (6 g/L) and as in Experiments 1 and 2, a watering can was used to apply 2.5 L of solute onto a plot. One hour after dye application, the soil was excavated to a depth of 30 cm in 1 cm thin increments and the soil was visually observed and photographed.

To determine the soil gravimetric water content, 5 g of field moist soil was dried at 105°C for 24 h. For the determination of the soil Br^−^ concentration, 3 g of field moist soil was extracted with 20 mL distilled water: soil and water were shaken in a Falcon tube (60 min), then centrifuged (3300 rotations/min, 20 min) prior to filtering through a glass fiber syringe filter (pore size of 0.45 μm). The filtered extracts were analyzed for Br^−^ on an Ion‐Chromatograph (Dionex ICS‐2100, ThermoFisher Scientific). The detection limit for Br^−^ was 20 μg/L in the water extract (0.133 mg kg^−1^).

The observed Br^−^ tracer distribution was used to determine the theoretical distribution and enrichment of isotopic tracers (^15^N as either NH_4_^+^ or NO_3_^−^, and ^18^O in H_2_O). It was assumed that the isotopic tracer solutions would migrate through the soil in a similar manner to the Br^−^ tracer solution (this is probably an overestimation for NH_4_^+^). By calculating the amount of tracer solution that reached a given soil depth, based on variations in Br^−^ recovery, we calculated the isotopic distribution in the soil. We assumed the tracer solution volume was equivalent to the Br^−^ solution applied (2.5 L); with 0.912 g N tracer L^−1^ and an enrichment of 60 at% ^15^N, equivalent to 40 kg N ha^−1^.^16^ By correcting for antecedent NH_4_^+^ and NO_3_^−^ dilution already in the soil, with natural abundance values of 0.385 and 0.380 at%, respectively, the theoretical isotopic ^15^N enrichments (at%) were calculated. The method was also used in an analogous way for calculating the potential ^18^O enrichment after application of H_2_^18^O with an original enrichment of 10 at%.

### 2.1 Laboratory experiment

Since the syringes were directly clogged during the test and thus, an application of the tracer was not possible with this method in this field, application with cannulas was further tested in the laboratory in Germany. One intact soil block (loamy sand) of 0.3 m × 0.3 m (depth 0.25 m) was extracted from ungrazed grassland at the experimental station of the University of Rostock. In the laboratory, this block was further cut into four blocks of 0.15 m × 0.15 m. Four cannulas were constructed from 10.3 cm long stainless‐steel tubes (outer diameter 3 mm, inner diameter 2 mm) closed and formed to a tip at one end. In each tube, eight holes were drilled with a longitudinal distance of 1.25 cm and a 90° turn between holes to optimize distribution (diameter of 2 mm for the top four holes and 1.5 mm for the four holes close to the tip).

All four cannulas were attached in a 7.5 cm square to the bottom of a sealed aluminum chamber (14.0 × 10.3 × 7.2 cm length × width × height), which served as a combined reservoir. To avoid blockage of the cannulas during insertion, compressed air was applied with an air gun attached to the top of the chamber. This might change structure and gas diffusivity of the soil. The chamber was aligned horizontally using a spirit level to ensure the same overhead for each cannula.

After insertion, 200 mL of stirred TiO_2_‐suspension (8.8 L/m²) was added to the chamber to trace infiltrated water by coloration.^21^ One soil block with four infiltration points each was evaluated after 30, 60, 90, and 120 min, respectively, to check for a temporal effect of the infiltration. To this end, each soil block was cut along a line directly connecting two cannulas as well as half way in between these two cuts.

As the infiltration into the soil was not homogeneous, the flow characteristics through the infiltration system and the newly designed cannulas was further tested using individual 45 mL pots for each cannula. Seven tests were performed using three different materials inside the pots to provide increasing flow resistance without clogging of needles (empty pots < cotton wool < sand). The cotton wool (∼2 g) was pushed into the pots by hand, the sand (dry, Ø 2 mm) was slightly compressed by tapping the pots on a table. Each material test was carried out with water and with TiO_2_‐suspension, except sand that was only tested with water. After applying 100 mL of solution, time for infiltration was measured and the amount of solution reaching each pot was measured gravimetrically. Each test was repeated three times for each material. Values of the outflow per pot were normalized by calculating their percentage shares of the total outflow volume of each repetition.

### 2.2 Statistics

For the measured variables, means and SDs were calculated for each soil depth. Furthermore, coefficients of variation were calculated for the horizontal as well as for the vertical resolution of selected variables within the soil. ANOVA was used to check for treatment effects and for differences in variables with soil depth (α ≤ .05). Data were tested for normality using the Shapiro‐Wilk test. If the requirements for ANOVA were not fulfilled, the Kruskal‐Wallis test was performed to determine treatment effects. The Tukey, Holm‐Sidák, and Dunn's test were used as post hoc tests. Statistical analyses were performed with SigmaPlot 13.0.

Concerning the flow characteristics, test means and SDs of the normalized outflow were calculated.

## 3 RESULTS

### 3.1 Field experiment

The watering can treatment had the shortest application time. Here, the total solution volume of 2.5 L was applied within 1 min (10 mm/min). Since the solution could be applied most quickly, this treatment also had the greatest potential for run‐off. For the application by sprayer, 3‐4 min per plot were required for solution application.

The treatment with the syringes was not successful under the given soil conditions, as the cannulas were blocked directly upon insertion into the soil. Even pre‐drilling the holes for the syringes did not lead to any further progress. In order to apply the volume of two syringes to the soil, about 10 min were needed. As this was not feasible under field conditions for larger areas, the treatment was abandoned. An experiment with syringes was instead performed in the laboratory to determine how homogeneously the tracer solution is distributed by syringe application (see below). The following field results only refer to the watering can and sprayer treatments.

#### 3.1.1 Soil water content dynamics

**FIGURE 1:**
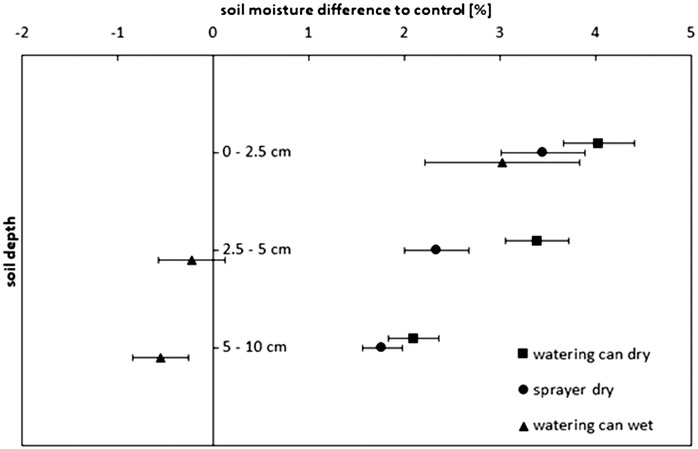
Comparison of the difference in gravimetric soil moisture content relative to the control treatment (%) over depth following Br^−^ solution application by watering can (square, Experiment 1), or sprayer (circle, Experiment 1) to dry soil, and by watering can to wet soil (triangle, Experiment 2). Data points are means (n = 4) ± standard errors

In Experiment 1, delivering the Br^−^ tracer solution using either the watering can or sprayer treatments increased the soil moisture relative to the control (Figure 1). After applying the Br^−^ tracer solution to dry soil, there was a slight, but significantly, larger soil moisture content when using the watering can compared with the sprayer (*P *= .047), when averaged over all depths. When comparing the watering can and sprayer treatments at individual depths, there were no significant treatment effects on soil moisture at depths 1 and 3 (*P *= .152*; P *= .344, respectively). However, in depth 2, application by watering can led to significantly wetter soil than with the sprayer (*P *= .030). Variation in soil moisture decreased with depth. The largest gravimetric water content, 30‐31%, was measured in depth 1: this held 3.5‐4.0% more water than the control (Figure 1). This difference decreased at depth 2 (2.3‐3.4%) and further at depth 3 (1.8‐2.1%). If the complete tracer solution had been recovered, the increase in soil moisture in contrast to the control would be 5.0‐5.5%, 3.6‐4.3%, and 2.6‐2.8% in depths 1 to 3, respectively.

The antecedent soil moisture had a significant effect (*P* ≤ .001) on changes in gravimetric soil water content caused by tracer application with watering can (Figure 1). This was especially remarkable in soil depths 2 and 3: following solution application by watering can to the wet soil, there was no increase in soil water content compared to the control; increases only occurred when solution was applied to dry soil.

In Experiment 1, the increase in soil moisture content following solution application to dry soil in the watering can treatment accounted for a total of 2 ± 1.3 L (80% ± 52%; here and in the following: means ± standard deviation) of the applied solution, corresponding to a loss of ∼0.5 L solution. On the plots where the sprayer was used, 1.6 ± 1.3 L (64 ± 52%) was accounted for, equivalent to a doubling of the loss compared to the watering can application. In Experiment 2, based on the increase in soil moisture, applying the solution to the wet soil resulted in only 0.324 ± 1.9 L (13 ± 76%) of the solution being recovered, with the largest recovery in the top soil layer (0.309 L).

#### 3.1.2 Bromide concentration

**FIGURE 2:**
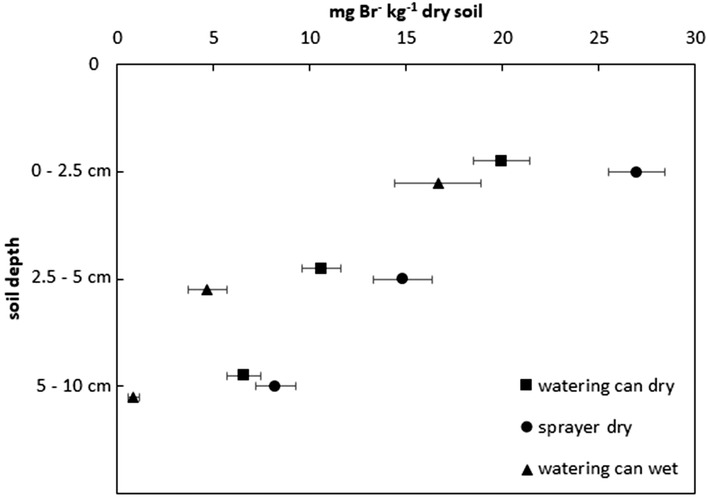
Comparison of Br^−^ concentration relative to control treatments (mg kg^−1^) over depth after tracer application by watering can (square) or sprayer (circle) to dry soil (Experiment 1) and by watering can (triangle) to wet soil (Experiment 2). Shown are means (n = 4) ± standard errors

In the control treatment, soil Br^−^ concentrations averaged 0.941 ± 0.081 mg Br^−^ kg^−1^. Elevated soil Br^−^ concentrations were found in all depths and treatments where Br^−^ was applied (Figure 2). When applied to dry soil, there was a significant difference (*P *= .016) between watering can and sprayer applications with, on average over all depths, 12 and 17 mg Br^−^ kg^−1^, respectively. When comparing the effect of the application treatments at individual depths, soil Br^−^ concentrations were only different at depths 1 and 2 (*P *= .001; *P *= .017, respectively). The largest differences between applications were observed in depth 1: the sprayer application led to a Br^−^ concentration of 27 mg Br^−^ kg^−1^, whereas the watering can treatment had a concentration of 20 mg Br^−^ kg^−1^ (Figure 2). At soil depth 2, the concentrations had declined by ca. 50% and ranged from 11 mg kg^−1^ (watering can) to 15 mg Br^−^ kg^−1^ (sprayer). In soil depths 1 and 2, both treatments had relatively large SDs, with those in the sprayer treatment larger than from the watering can (up to ± 6 mg Br^−^ kg^−1^). In soil depth 3, there was no significant difference between the watering can and sprayer treatments, which averaged 7 mg Br^−^ kg^−1^ (*P *= .226).

Over all depths, application of Br^−^ solution by watering can to wet soil (Experiment 2) led to significantly smaller Br^−^ concentrations than application to dry soil (*P* ≤ .001, Figure 2). The difference was not significant at depth 1 (*P *= .058). Here, the SD of Br^−^ concentrations in the wet soil was larger than at deeper depths. Also, the mean concentration decreased with depth, with no significant difference between the control and watering can application at depth 3 after Br^−^ application to wet soil (*P *= .066, 1.0 mg Br^−^ kg^−1^).

Overall, more Br^−^ was recovered in the plots where the solution was applied with the sprayer than with the watering can to dry soil. In Experiment 1, for the watering can treatment, 830 ± 330 mg of the 1.25 g Br^−^ applied (67 ± 26%) was recovered, while 1085 ± 391 mg of Br^−^ (87 ± 31%) was recovered in the plots with sprayer application. In Experiment 2, 490 ± 709 mg of Br^−^ (39 ± 57%) was recovered in the wet soil. Compared to the results of soil moisture, it was obvious that the watering can treatment in dry soil had the highest recovery of the applied water, but a lower Br^−^ recovery than the sprayer treatment.

The coefficients of variation for the Br^−^ concentrations with depth (vertical resolution) were consistently smaller than those for soil moisture (Table 1). The treatments applied to dry soil resulted in very similar coefficients of variation. The application by watering can to wet soil generated the largest coefficients of variation (Table 1). In the horizontal direction, the coefficients of variation were larger for soil moisture than for soil Br^−^ concentrations, with an exception being the deepest soil layer following applications to dry soil (data not shown). On wet soil, the horizontal coefficients of variation were overall very large. All horizontal variation coefficients increased in the Br^−^ method with depth, while they tended to decrease for soil moisture (data not shown).

**TABLE 1:** Coefficients of variation for the different treatments pooled over all depths (n = 12) (watering can on dry and wet soil, sprayer on dry soil) in vertical resolution

Treatment	Soil Moisture	Br^−^ Concentration
Watering can on dry soil	0.479	0.403
Sprayer on dry soil	0.402	0.355
Watering can on wet soil	2.546	0.599

**FIGURE 3:**
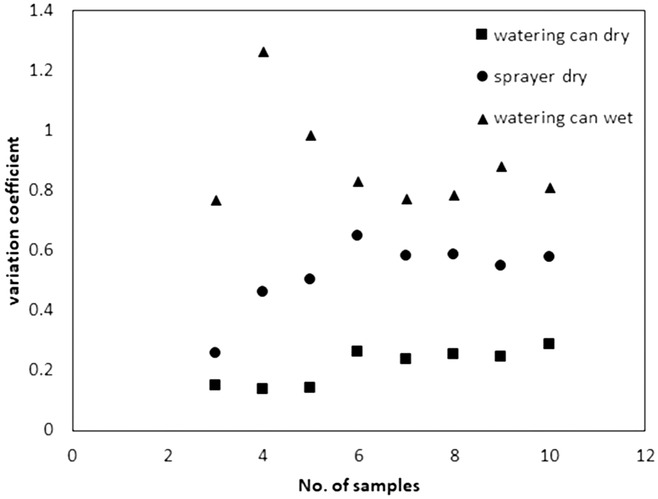
Variation coefficients depending on the number of soil samples. Shown are three different treatments (watering can and sprayer on dry soil, watering can on wet soil)

To determine homogeneity of distribution of tracer solution, we took 10 samples per soil depth in this experiment. Figure 3 shows the development of the coefficients of variation with the number of samples taken. The coefficients of variation of the watering can treatment on wet soil and the sprayer treatment on dry soil varied more with a smaller number of samples than those of the watering can treatment on dry soil. The largest coefficients of variation were found in the wet soil, whereas the smallest were observed in the watering can treatment on dry soil. From six to seven soil samples onward (per 0.25 m²), the coefficients of variation remained stable.

#### 3.1.3 Experiment with blue dye

**FIGURE 4:**
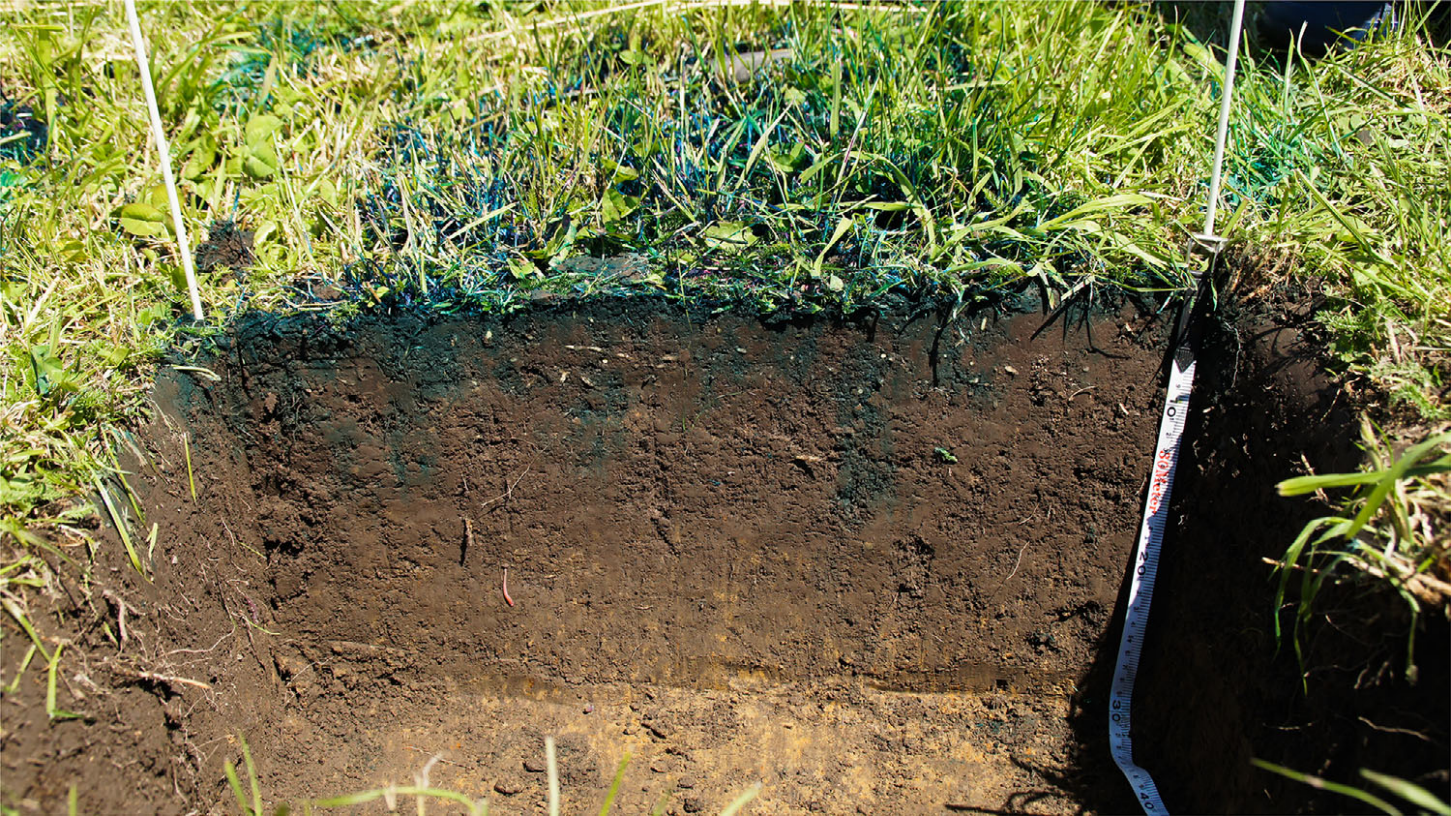
Photo of a cross section of a typical soil depth profile (depth = 30 cm) showing penetration of “brilliant blue” dye in wet soil

In Experiment 3, the blue dye provided clear visual evidence of run‐off, with dye found outside of the plot. The depth profiles clearly demonstrated that the tracer dye was not uniformly distributed (Figure 4), even though most of the tracer could be found in the upper 10 cm. In some places, the tracer could be observed at a depth >20 cm, especially in combination with earthworm burrows and roots.

#### 3.1.4 Theoretical isotopic tracer enrichments

**FIGURE 5:**
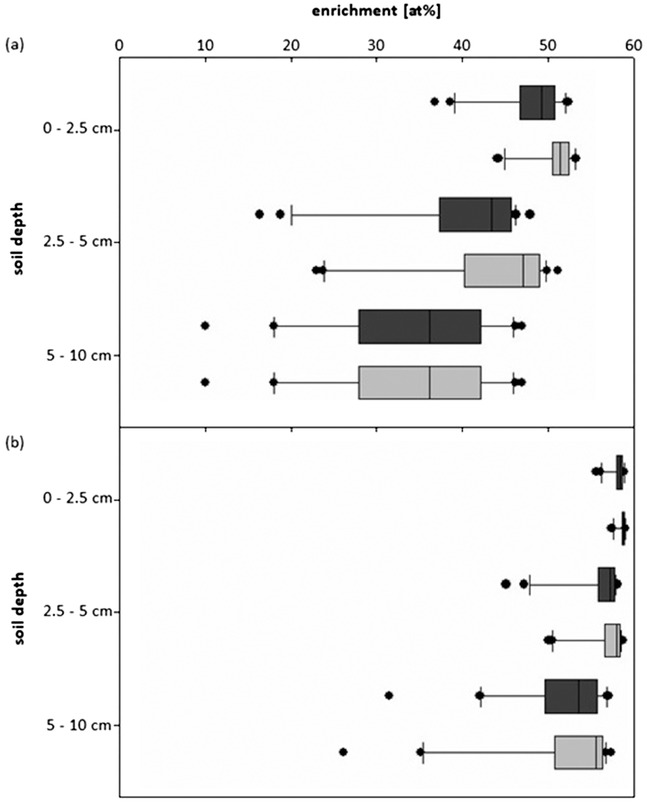
Resulting theoretical enrichments (at%) of ^15^NH_4_^+^ (a) and ^15^NO_3_^−^ (b) for the watering can (dark grey) and the sprayer (light grey) treatments on dry soil for three soil depths, calculated based on the distribution of Br^−^ tracer solution assuming an application of 60 at%

The calculated enrichments of ^15^NH_4_^+^ generally showed a slight decrease with increasing soil depth (Figure 5A). In the upper 2.5 cm, the ^15^NH_4_^+^ tracer enrichment was diluted to a median value of 51 at% in the sprayer treatment, with values ranging from 44 to 53 at%. For all soil depths, the values above the median showed rather little variation, whereas those below varied over a range of 20 at%. In the upper 2.5 cm, variation was mostly found in the lowest quartile, whereas in 5‐10 cm, the variation was mainly in the quartile below the median. In the deepest soil depth, both treatments showed a median potential enrichment of 34 at%. Overall, the potential enrichments with the sprayer treatments were slightly but not significantly larger than with the watering can (*P* = .097).

The calculated potential enrichments for ^15^NO_3_^−^ showed a very similar pattern (Figure 5B), but the data were less dispersed (median range 56‐59 at%). The theoretical dilution of the ^15^NO_3_^−^ tracer was not as strong as with ^15^NH_4_^+^, as the antecedent NO_3_^−^ concentration was very small. The median enrichment was at 58 at% in the upper 2.5 cm and even the lowest soil depth reached potential enrichments of more than 50 at%, with large variations. Again, the distribution of data was negatively skewed at all three depths and no significant differences were found (*P* = .699). The calculated theoretical enrichments of NO_3_^−^ in the soil did not differ between tracer application methods.

**FIGURE 6:**
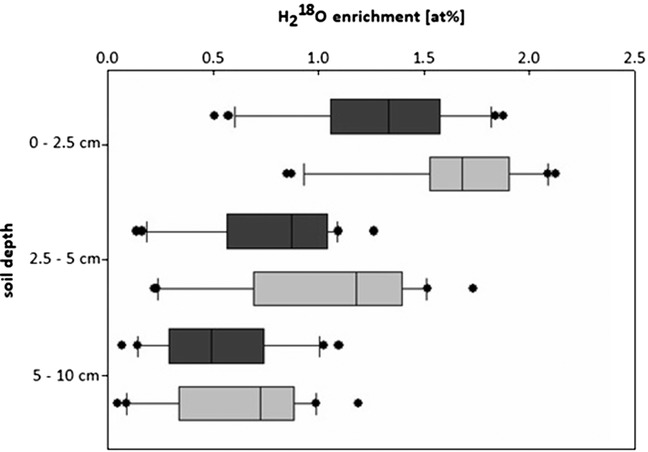
Resulting theoretical enrichments of H_2_^18^O (at%) for the watering can (dark grey) and the sprayer (light grey) treatments on dry soil for three soil depths, calculated based on the distribution of Br^−^ tracer solution assuming an application of 10 at%

In the third theoretical scenario, with H_2_^18^O, the calculated potential enrichment also decreased over soil depth (Figure 6). Due to the antecedent soil moisture content, the dilution was larger than for ^15^NO_3_^−^ and ^15^NH_4_^+^, being, on average, a 1/9th dilution. The largest calculated ^18^O enrichment was just 2.2 at%, and the smallest only 0.2 at%. As for the ^15^N enrichments, the potential calculated ^18^O enrichments with the sprayer treatment were slightly larger than with the watering can, however, the different application methods did not show a significant difference (*P* = .232).

### 3.2 Laboratory experiment with multi‐hole cannulas

**FIGURE 7:**
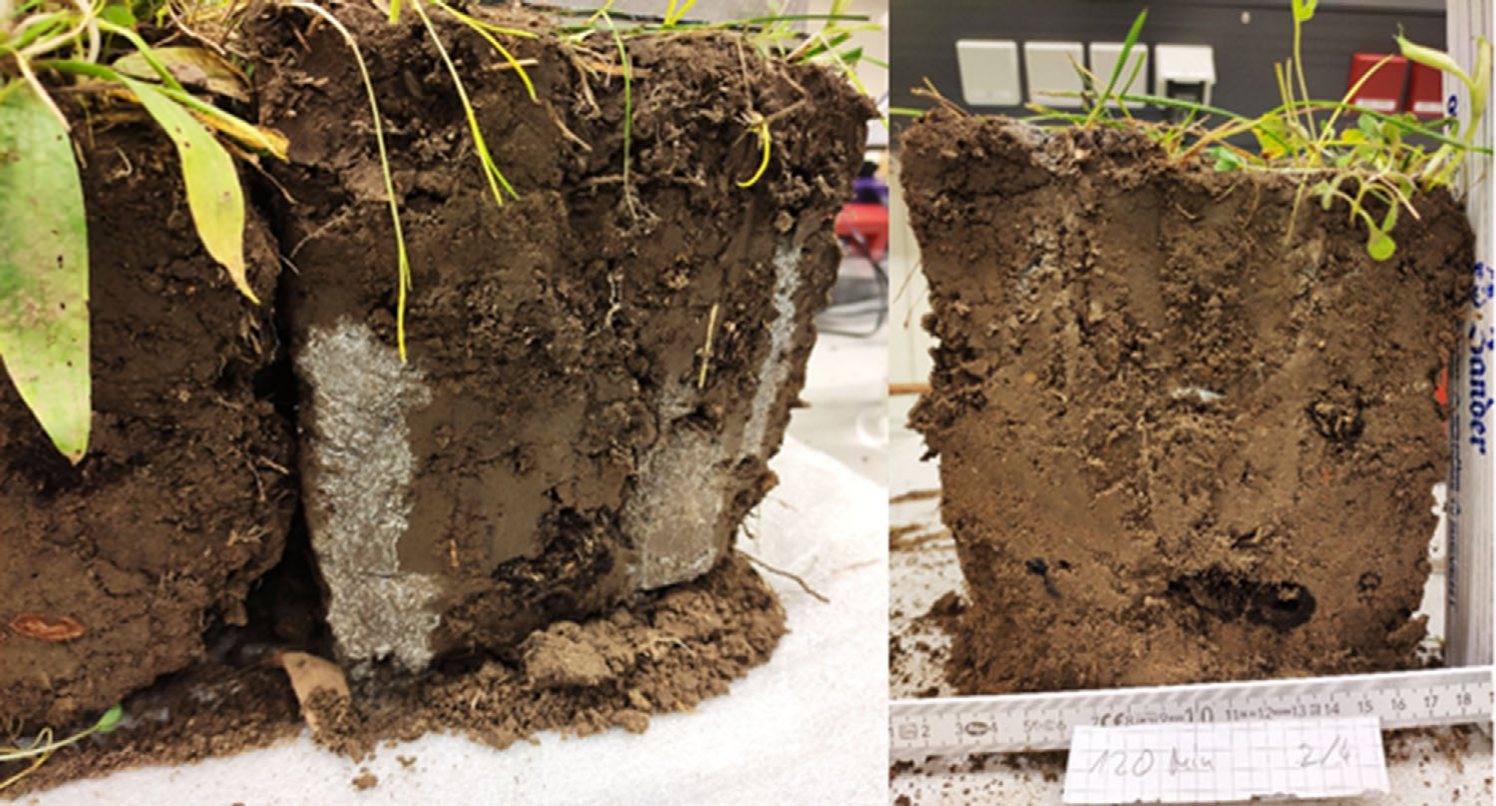
Left: Intact soil block (15 cm × 15 cm) after infiltration of TiO_2_‐suspension with three white colored areas marking points of extensive outflow of TiO_2_‐suspension from the sides of the block. Right: Cut though the infiltration points 2 and 4 showing only minor white coloration in the areas of the cannulas and one intensely colored hot spot (center of the soil block) that was identified as earthworm hole

Only seconds after starting infiltration, substantial amounts of TiO_2_‐suspension were flowing out of macropores from the sides and bottom of the soil blocks. The soil profiles matched this observation: Only very small or no visible coloration by TiO_2_ in the area close to the cannulas was recognizable (Figure 7). Concurrently, well‐colored hotspots were identified along earthworm holes and decaying roots.

**FIGURE 8:**
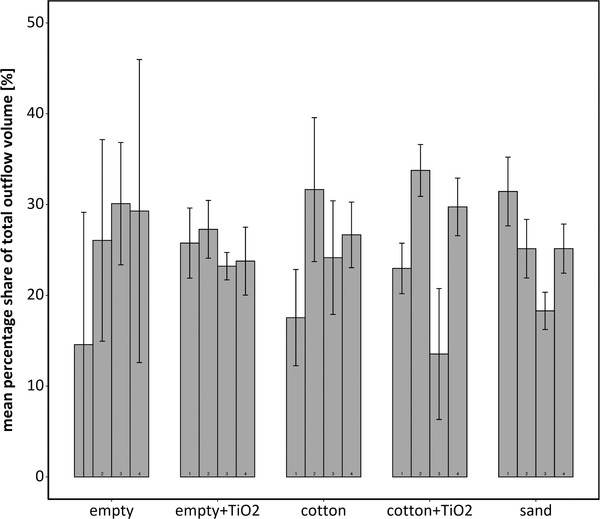
Mean percentage share of individual cannulas (1‐4) of the total outflow volume of the respective repetition (n = 3) ± standard errors

The test of the flow characteristics showed large variations in outflow volume of individual cannulas (Figure 8), especially when testing with water in empty pots (0.3‐46% share of total volume per cannula). Variations in outflow volume per cannula resembled the variations among all cannulas. There was no consistent effect of cannula, water versus TiO_2_ or material in the pots on the amount of outflow per cannula. The duration of infiltration was similar for empty pots and those filled with cotton wool (47‐67 s). With sand in the pots, the first repetition with water took 76 s and fully infiltrated the volume of the 45 mL pots. In the second and third repetition, water infiltrated in the top of the pots and about one‐third of the water flowed to the surface and spilled over the pots’ edges.

## 4 DISCUSSION

In this study, we compared the practicality of and resulting homogeneity after applying a tracer solution to a pasture soil, in situ, using either a sprayer, watering can or syringe. Under the soil conditions at our test site, the cannulas of the used syringes were blocked immediately, even in pre‐drilled holes. Of course, pre‐drilling should be avoided as the solution would otherwise follow this macropore. Nevertheless, there are other soil conditions where syringe application is feasible. As also the tests with side‐port cannulas in another soil and the very standardized laboratory setups showed very large variation among cannulas and preferential flow though macropores into deeper soil layers, we also expect a heterogeneous distribution of water and any solved tracers. The only benefit over the other application methods might be less runoff. Basically, to avoid preferential flow, it seems that the hydraulic head has to be minimized. Additionally, it has to be verified if flushing the soil with compressed air might change structure and gas diffusion of the soil.

As the outflow volume of the multi‐hole cannulas strongly fluctuated among cannulas but also per cannula among repetitions, this was probably not caused by design or the chamber not being perfectly horizontal, but by reasons concerning the flow characteristics of the cannulas themselves. An assumption is that small bubbles inside the cannulas cause a strong resistance due to surface tension in a small diameter tube. Although equipping each cannula with its own reservoir might somewhat reduce heterogeneities, blocking of cannulas or sections thereof by air bubbles would still be possible. Therefore, the concept of multi‐hole soil cannulas does not appear very promising. Using single or dual‐hole cannulas with their own reservoir and used in several steps in multiple depths may avoid problems from blocked sections, but the biggest problem caused by preferential flow through macropores will likely persist. Slowing the flow through the cannulas might mitigate the flow through macropores and lead to a better distribution. We therefore suggest an application method that is avoiding overhead pressure and is utilizing the capillary forces of the soil to achieve a homogeneous distribution of dissolved substances (tracers).

In line with the first hypothesis, there were differences among the application treatments using watering can, sprayer, or syringes concerning the time needed for application as well as the homogeneity of application. The watering can allowed the fastest application, requiring 1 min per plot, whereas the sprayer application required 3‐4 min. The difference in these application times would be significant if the first soil samples were required to be taken immediately after application of solutions and if resources for sampling were limited. Large standard errors for mean Br^−^ concentrations (Figures 1 and 2) and horizontal variation coefficients (data not shown), as well as decreasing tracer concentrations with increasing depths (Figure 1) demonstrate that the Br^−^ tracer was not homogeneously distributed. The vertical gradients will change over time due to diffusion (we took samples one hour after application) and leaching following precipitation events. As the used soil was under permanent grassland, the formation of macropores is favored,^22^ increasing the potential for preferential flow.^23^ This influences both the distribution of the tracer upon application and the changes in distribution after precipitation events.

Although we took great care when applying tracer solution, some horizontal inhomogeneity might have been caused by the manual application. To avoid this, tracers might be applied mechanically, for example with a sprinkler system with a constant flow. This would enable a more uniform application of the solution and could contribute to minimizing errors. However, this would also not prevent preferential flow of solution, for example, along roots or earthworm burrows (Figure 4).

Contrary to our hypothesis, there was no significant correlation between the measured Br^−^ and the observed change in moisture caused by the treatment (*P* = .879). There were, however, clearly significant correlations between gravimetric moisture content and measured Br^−^ concentration (*P* ≤ .001). The poorer correlation between relative change in soil moisture between treated and control plots and soil Br^−^ concentrations could have been due to heterogeneities in soil moisture distribution, causing initial differences between measured control and treated plots.

The results show that in general, the distribution of the Br^−^ solution was more homogeneous when applied by sprayer than by watering can. The coefficients of variation were smaller than those of the watering can treatment, both overall and in depths 1 and 2, and recovery of tracer was larger. Blue dye application also clearly showed that run‐off to outside the plot could occur when using the watering can. We assume that run‐off was larger with the watering can treatment than the sprayer and therefore, more Br^−^ was deposited in the soil inside the plot with the sprayer treatment. In the watering can treatment, ∼67% of the Br^−^ was recovered versus 82% in the sprayer treatment. Losses might have occurred due to either run‐off or penetration to deeper layers. The faster application in the watering can treatment will have resulted in a larger hydraulic head, potentially making it possible for the solution to reach deeper soil layers more quickly. Elrick and Parkin^24^ suggested that a larger hydraulic head would lead to a larger flow rate into the soil. This could cause greater macropore flow and consequently more leaching of solution, increasing the tracer solution loss. In the dye experiment, the tracer was found below 20 cm in combination with earthworm borrows and roots. We did not find significant differences in Br^−^ concentration or soil moisture changes between sprayer and watering can application in the deepest soil layer of the dry soil studied here (Figures 1 and 2). Therefore, the potentially larger hydraulic head caused by the watering can application apparently did not lead to deeper soil penetration, but potentially more run‐off. Another factor potentially affecting infiltration of tracer solution is the presence of plants. The vegetation at our test site consisted mainly of grasses, which can intercept tracer solution, especially at lower precipitation intensity^25^ as in the sprayer treatment. For further studies, vegetation should be considered, as leaf surfaces, for example, intercept more solution and thus reduce infiltration into a soil, whereas surfaces with less vegetation would also show less interception and therefore, a larger soil infiltration.

As hypothesized, the wetter the soil, the less Br^−^ was recovered. The upper soil depth showed a distinct change in soil moisture and consequently an increase in Br^−^ concentrations. Since hardly any tracer solution infiltrated the deeper soil depths 2 and 3 when applied to the wetter soil, the Br^−^ concentration here was also clearly smaller. Timlin et al^26^ suggested that in dry soil, the solution moves with large pressure gradients into the smaller pores. These smaller pores drain more slowly than larger pores and therefore, the tracer remains longer and vertical transport is reduced. Also Heathman et al^27^ showed that in soils with dry aggregates, the downward movement of Br^−^ tracer was delayed.

Given that the Br^−^ recoveries were larger and coefficients of variation were significantly smaller in the dry soil, it is clear that isotopic tracer should be applied to dry soil if possible, and that a spray application is better suited. However, in order to investigate denitrification, experiments are commonly performed on soils with larger water contents. In this case, the results indicate that extra water should be added with the tracer in order to generate denitrifying conditions, rather than adding tracer to a wetted soil. To assist in interpretation of the results, the volume of soil affected by tracer additions must also be identified using Br^−^ or dye.

The coefficient of variation of measured variables (soil water, soil Br^−^ concentration) varied with sample number (Figure 3). It became stable when at least six or seven soil cores were taken from an area of 0.25 m², reflecting the inhomogeneity of the soil. An understanding of the potential variation due to sample numbers, as attained here, should also be a requirement for a field study.

Concerning theoretical isotopic enrichments, the SDs for ^15^NO_3_^−^ were much smaller than those for ^15^NH_4_^+^ since considerably less NO_3_^−^ than NH_4_^+^ was initially present in this soil, leading to less dilution. Thus, the homogeneity of tracer distribution depends – in addition to the application technique – also on soil parameters like the water content or the antecedent NH_4_^+^ and NO_3_^−^ concentrations.^28^ Although about 50% of the soil volume would have a ^15^N enrichment similar to that aimed at, the remaining 50% would have a reduced enrichment, especially where soil stocks dilute the tracer. Variations in enrichments of mineral N will lead to equivalent variations in the enrichment of N_2_O and in the following also in the calculated processes responsible for N_2_O emissions. Of course, ^18^O‐H_2_O can be expected to be more diluted due to water already present in the soil. On average, it was calculated to reach only 10% of the added enrichment. However, the heterogeneity was calculated to be much less than for ^15^N tracers. Importantly, the variation in calculated potential enrichments cannot be decreased by increasing the enrichment of the used tracer, but by adding more tracer to the soil (Table 2).

**TABLE 2:** Sensitivity analysis of inhomogeneity of isotopic tracer application: Shown are standard errors (S. E.) depending on (a) variations in the enrichment of isotopic tracer and (b) variations of the amount of tracer applied

(a) Depending on enrichment (0.912 g/L applied)					
	60 at%	70 at%	80 at%	90 at%	100 at%
S. E.	0.559	0.652	0.745	0.838	0.931

(b) Depending on the tracer amount (60 at% applied)					
	0.912 g/L	1.2 g/L	1.5 g/L	1.7 g/L	2 g/L
S. E.	0.559	0.467	0.397	0.361	0.318

In summary, this study shows that there are clear differences in homogeneity of tracer application and time needed among methods. Neither application method, watering can, sprayer nor injection led to a homogeneous distribution of tracer due mainly to macro‐pore flow and dilution as well as needle clogging by clay or air bubbles for injection. Spraying led to slightly better results than the other methods. However, so far, the tested methods do not provide a sound basis for differentiating soil processes leading to N_2_O production.

To improve homogeneity, we suggest to reduce the hydraulic head during application, but to use larger volumes of isotopic tracers. Applying tracers to dry soils should be preferred. Possibility of injection needs to be tested in the given soil. Run‐off, or overland flow, and leaching to deeper soil layers needs to be assessed using dyes and Br^−^ tracer. Then, isotopic values might be corrected for heterogeneity of tracer application.
